# Oxygen Is an Ambivalent Factor for the Differentiation of Human Pluripotent Stem Cells in Cardiac 2D Monolayer and 3D Cardiac Spheroids

**DOI:** 10.3390/ijms22020662

**Published:** 2021-01-11

**Authors:** Monia Souidi, Yvonne Sleiman, Ivana Acimovic, Jan Pribyl, Azzouz Charrabi, Volker Baecker, Valerie Scheuermann, Martin Pesl, Sarka Jelinkova, Petr Skladal, Petr Dvorak, Alain Lacampagne, Vladimir Rotrekl, Albano C. Meli

**Affiliations:** 1 PhyMedExp, INSERM, University of Montpellier, CNRS, 34000 Montpellier, France; moniasouidi@yahoo.com (M.S.); yvonne.sleiman@umontpellier.fr (Y.S.); acimovic.ivana@gmail.com (I.A.); azzouz.charrabi@inserm.fr (A.C.); valerie.scheuermann@inserm.fr (V.S.); alain.lacampagne@inserm.fr (A.L.); 2 Department of Biology, Faculty of Medicine, Masaryk University, 62500 Brno, Czech Republic; sarka.jelinkova89@gmail.com (S.J.); pdvorak@med.muni.cz (P.D.); vrotrekl@med.muni.cz (V.R.); 3 CEITEC, Masaryk University, 62500 Brno, Czech Republic; pribyl@nanobio.cz (J.P.); skladal@chemi.muni.cz (P.S.); 4 Montpellier Ressources Imagerie, BioCampus Montpellier, CNRS, INSERM, University of Montpellier, 34000 Montpellier, France; volker.baecker@mri.cnrs.fr; 5 International Clinical Research Center, St. Anne’s University Hospital Brno, 65691 Brno, Czech Republic; 6 First Department of Internal Medicine/Cardioangiology, St. Anne’s Hospital, Masaryk University, 65691 Brno, Czech Republic

**Keywords:** hPSC-derived cardiomyocytes, cardiac spheroids, embryoid bodies, 2D-monolayer, oxygen exposure, intracellular calcium handling, mitochondrial oxygen consumption, contractile properties

## Abstract

Numerous protocols of cardiac differentiation have been established by essentially focusing on specific growth factors on human pluripotent stem cell (hPSC) differentiation efficiency. However, the optimal environmental factors to obtain cardiac myocytes in network are still unclear. The mesoderm germ layer differentiation is known to be enhanced by low oxygen exposure. Here, we hypothesized that low oxygen exposure enhances the molecular and functional maturity of the cardiomyocytes. We aimed at comparing the molecular and functional consequences of low (5% O_2_ or LOE) and high oxygen exposure (21% O_2_ or HOE) on cardiac differentiation of hPSCs in 2D- and 3D-based protocols. hPSC-CMs were differentiated through both the 2D (monolayer) and 3D (embryoid body) protocols using several lines. Cardiac marker expression and cell morphology were assessed. The mitochondrial localization and metabolic properties were evaluated. The intracellular Ca^2+^ handling and contractile properties were also monitored. The 2D cardiac monolayer can only be differentiated in HOE. The 3D cardiac spheroids containing hPSC-CMs in LOE further exhibited cardiac markers, hypertrophy, steadier SR Ca^2+^ release properties revealing a better SR Ca^2+^ handling, and enhanced contractile force. Preserved distribution of mitochondria and similar oxygen consumption by the mitochondrial respiratory chain complexes were also observed. Our results brought evidences that LOE is moderately beneficial for the 3D cardiac spheroids with hPSC-CMs exhibiting further maturity. In contrast, the 2D cardiac monolayers strictly require HOE.

## 1. Introduction

The study of human development and disease is too often inaccurately modeled in animals. Human pluripotent stem cells (hPSC), including human embryonic stem cells (hESC) and, more recently, human induced pluripotent stem cells (hiPSC), theoretically have the ability to self-renew and differentiate in any cell type, providing an excellent tool for research [1]. These cells allow the development of organoid systems that exhibit some major features of a real human organ, such as intestine, liver or heart. Such exciting advancement in stem cell research is currently of very high interest. Consequently, finding the optimal conditions to form these in vitro cardiomyocyte networks is crucial and the differentiation protocols must be developed by improving each experimental parameter for the highest efficacy in forming the desired cell type. In mammals, oxygen (O_2_) exposure plays a key role in stem cells and during the embryogenesis [2]. Hence, it has been shown that the mammalian embryo preferentially grows in an environment comprising 1 to 10% O_2_ [3]. In particular, evidence indicates that low O_2_ exposure (LOE typically known as 5% O_2_, 5% CO_2_) is critical for maintaining normal cardiomyogenesis in the early stage of embryonic development that leads eventually to the formation of blood vessels and the mammalian heart [2,3]. Moreover, the cardiac progenitor cells have been found to be preferentially located in LOE niches [4,5]. Several 3D spherical embryoid bodies (EBs) and 2D monolayer culture protocols have been established in the last decade to obtain cardiac myocytes in network [1,6]. However, while LOE seems to be crucial for in vivo normal cardiac development, it is still unclear whether it is needed to optimize the 2D and 3D in vitro cardiac protocols. In fact, some protocols differentiate the hPSC-CMs under high oxygen exposure (HOE typically known as 21% O_2_, 5% CO_2_), while some do so under LOE [1].

It has been shown that LOE promotes the expression of mesodermal genes in hPSC [7]. We evaluated the gene expression of hypoxia inducible factor 1 alpha (*HIF1A*) and found no effect of LOE. One explanation might be related to the age of the cardiac spheroids first incubated under LOE for 12 days followed by HOE. By using mouse embryos and embryonic stem cells, it was shown that HIF-1α is crucial for normal cardiomyogenesis, where it leads to a better formation and differentiation of cardiac cells [8]. Deficiency of HIF-1α induces cardiac malformations including dysfunctional vasculogenesis, angiogenesis and morphological defects such as the loss of formation of cardiac chambers [9,10]. In fact, the hypoxic conditions enhance the expression of myocyte enhancer factor 2C (Mef2C), T-Box Transcription Factor 5 (Tbx5) and titin, one of the most important cardiac transcriptional factors that control the myofibrillogenesis [9]. In contrast, Medley et al. showed that LOE (2% O_2_, 5% CO_2_) impairs mouse induced pluripotent stem cells (miPSC) to differentiate in cardiomyocytes and activates canonical Wnt signaling pathway in undifferentiated miPSC [11]. Taken together, these studies have given an unclear conclusion regarding the role of oxygen exposure on the human cardiac differentiation in the dish. In particular, these studies strikingly reported different reactions of murine and hPSC to oxygen exposure upon differentiation. Nonetheless, its role in molecular and functional properties of hPSC-CMs at a later stage remains unclear.

In the present study, we aimed at evaluating the impact of oxygen exposure (LOE vs. HOE) by using 2 of the most commonly used protocols of cardiac differentiation in vitro [1,12,13,14,15,16]. We evaluated some of the major molecular and functional properties of the hPSC-CMs obtained from the 2D (sandwich-matrix monolayer) and 3D (EB-based) protocols at several stages.

## 2. Results

### 2.1. LOE Abolishes the Differentiation of hPSC in 2D Cardiac Monolayers But Not in 3D Cardiac Spheroids

**Figure 1:**
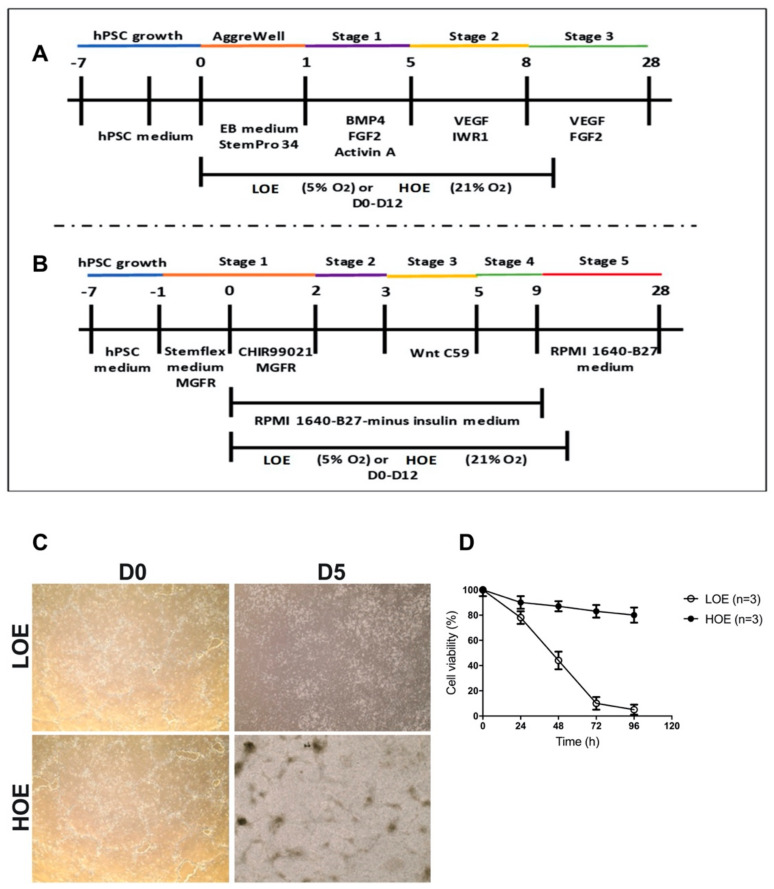
Timeline of the differentiation protocols of hPSCs. (**A**) 3D-based protocol for the formation of cardiac spheroids using AggreWell plates. (**B**) 2D-based protocol for the monolayer formation via the sandwich-based protocol. (**C**) Images illustrating the state of the hPSC at day 0 and 5 upon cardiac differentiation via 2D monolayer protocol under LOE and HOE. The acquisition was performed using EVOS XL Core Imaging System microscopy (magnification x4). (**D**) Cell viability in 2D cardiac monolayer (using hiPSC UB47 line) differentiated in LOE (white dot plots) and HOE (black dot plots) conditions. The number of experiments is 3 independent biological replicates. Data are shown as mean ± SEM. Significance was calculated by 2-way ANOVA, Bonferroni’s multiple comparison.

To supplement the previous studies achieved on early cardiac differentiation [8,11], we first determined whether the oxygen exposure (LOE vs. HOE, Figure 1A,B) leads to late cardiac differentiation in 2D human monolayers. However, upon 5 independent attempts, we could not maintain the hPSC in monolayer under LOE as the cells quickly died between 72 and 96 h (Figure 1C,D). In comparison, HOE allowed maintaining the hPSC upon cardiac differentiation up to day 30. We then applied LOE and HOE to differentiate hPSC in 3D cardiac spheroids.

### 2.2. Oxygen Exposure Does Not Affect the Mitochondrial Distribution and Function in 3D Cardiac Spheroids

**Figure 2:**
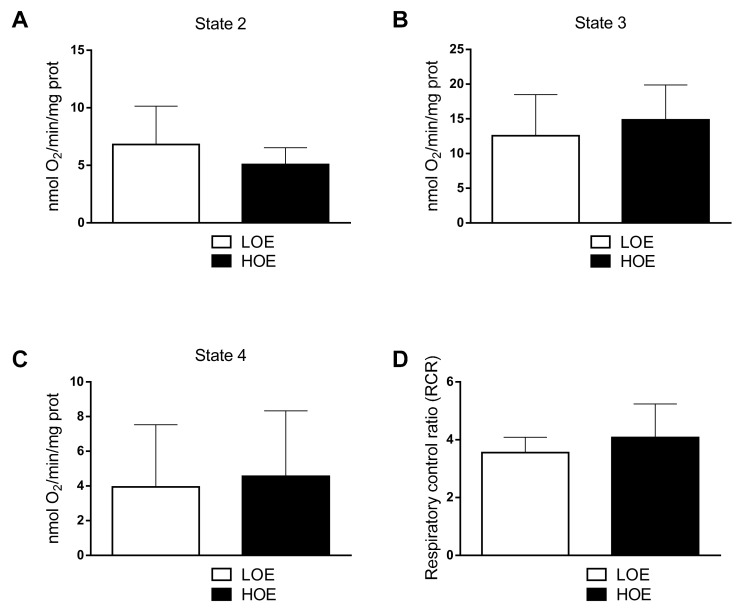
The mitochondrial O_2_ consumption is similar in both 3D cardiac spheroids differentiated under LOE and HOE conditions. The mitochondrial O_2_ consumption in 12 days old hPSC-CMs (using hiPSC UEFhfiPS1.4 and UB47 lines) differentiated in LOE and HOE conditions was evaluated using the O_2_-k oxygraph (Oroboros Instruments). (**A**) State-2 respiration in hPSC-CMs differentiated under LOE (white bars) and HOE (black bars) conditions. (**B**) State-3 respiration in hPSC-CMs differentiated under LOE and HOE conditions. (**C**) State-4 respiration in hPSC-CMs differentiated under LOE and HOE conditions. (**D**) Respiratory control ratio (RCR) in hPSC-CMs differentiated under LOE and HOE conditions. The number of experiments is 3 independent biological replicates for each bar graph. Data are presented as mean ± SEM. Significance was calculated by Mann-Whitney test.

As a major component of the O_2_ exposure, we first investigated the impact of LOE and HOE on the mitochondrial metabolic properties of the hPSC-CMs at an early stage (stage 3, Figure 1A) when the 3D cardiac spheroids are being differentiated (12-day-old). We first hypothesized that HOE increases O_2_ consumption in hPSC-CMs when compared to LOE. Using 3 independent cardiac differentiation assays, we assessed the mitochondrial respiratory rate by means of high-resolution respirometry using an O_2_K-Oxygraph (Oroboros Instruments). Surprisingly, the O_2_ consumption was similar in both conditions in the rate of states 2, 3 and 4 (3.94 ± 2.07 nmol O_2_/min/mg protein for LOE vs. 4.56 ± 2.17 nmol O_2_/min/mg protein for HOE, *p* = 0.7) (Figure 2A–C). The respiratory control ratio (RCR) constitutes the ratio of mitochondrial respiration supporting ATP synthesis to that required to offset the proton leak and represents the mitochondrial coupling state (a high RCR implies that the mitochondria have a high capacity for substrate oxidation and ATP turnover and a low proton leak). Herein, no significant differences of the RCR between the two conditions were observed (ratio of 3.56 ± 0.52 for LOE vs. ratio of 4.08 ± 1.15 for HOE, *p* = 0.7) (Figure 2D).

**Figure 3:**
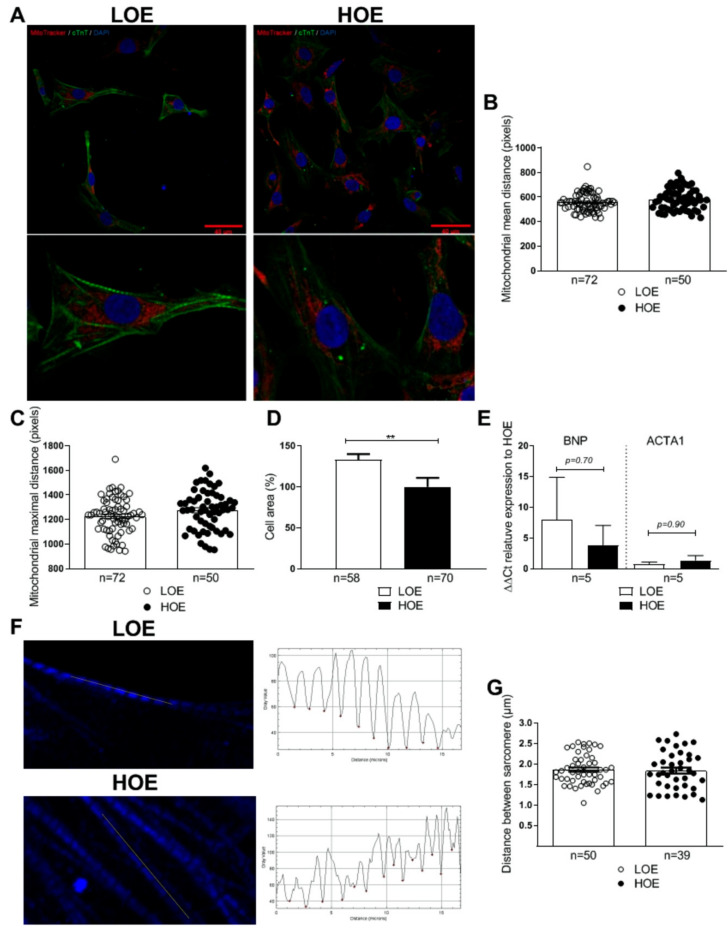
The mitochondrial distribution is similar in both 3D cardiac spheroids differentiated under LOE and HOE conditions. Mitochondrial localization and cell area in 25-day-old hPSC-CMs (using hiPSC UEFhfiPS1.4 and hESC CCTL12 lines) differentiated in LOE and HOE conditions were evaluated using MitoTracker dye. (**A**) Immunostaining showing the MitoTracker signal (red) and the cTnT staining (green) in 25-day-old hPSC-CMs obtained from 3D cardiac spheroids differentiated under LOE and HOE conditions. Nuclei are stained with DAPI (blue). Scale bars: 40 µm. (**B**) Mitochondrial mean distance in 25-day-old hPSC-CMs obtained from 3D cardiac spheroids differentiated under LOE (white dot plots) and HOE (black dot plots) conditions. (**C**) Mitochondrial maximal distance in 25-day-old hPSC-CMs obtained from 3D cardiac spheroids differentiated in LOE and HOE conditions. (**D**) Cell area of 25-day-old hPSC-CMs (using hiPSC UEFhfiPS1.4 and UB47 lines) obtained from 3D cardiac spheroids differentiated in LOE and HOE conditions. (**E**) ΔΔCt relative gene expression analysis by qRT-PCR in 25-day-old hPSC-CMs obtained from 3D cardiac spheroids differentiated in LOE (white bars) and HOE (black bars) conditions for BNP= natriuretic peptide B and ACTA1= actin alpha skeletal muscle 1. (**F**) Measurement of the sarcomere length of hPSC-CMs (using hiPSC UEFhfiPS1.4 and hESC CCTL12 lines) in the two differentiation protocols by tracing a line of 17 µM across the sarcomeres using an in-house developed macro on Fiji-Image J. Sarcomeric cTnT is stained in blue. Longitudinal plots were obtained through the translation of the fluorescence intensity across the line. (**G**) Distance between sarcomere in hPSC-CMs (using hiPSC UEFhfiPS1.4 and hESC CCTL12 lines) differentiated in LOE and HOE conditions. The number of cells evaluated varies from 39 to 72 for each dot plot from 3 independent biological replicates. Data are presented as mean ± SEM. Significance was calculated by Mann-Whitney test. **, *p* < 0.01.

It has been shown that adult CMs display subsarcolemmal and interfibrillar mitochondria when compared to neonatal CMs rich in perinuclear mitochondria [17]. Thus, we assessed whether O_2_ has an impact on the mitochondrial localization in 25-day-old hPSC-CMs in 3D cardiac spheroids. Using MitoTracker staining, we found no change in the mean distance from the nucleus (558.6 ± 8.72 pixels for LOE vs. 582.3 ± 11.32 pixels for HOE, *p* = 0.12) and in the maximal distance from the nucleus (1226 ± 17.47 pixels for LOE vs. 1276 ± 20.61 pixels for HOE, *p* = 0.06) (Figure 3A–C).

We then determined whether oxygen exposure during differentiation modulates the size of the hPSC-CMs by measuring the cell area. We found that LOE induces cellular hypertrophy and increased the cell area by 33% when compared to hPSC-CMs under HOE (133 ± 7%, *n* = 58 for LOE vs. 100 ± 11%, *n* = 70 for HOE, *p* < 0.01) (Figure 3D). We evaluated by qPCR the expression of 2 specific hypertrophic markers such as natriuretic peptide B (*BNP*) and actin alpha skeletal muscle 1 (*ACTA1*). No differences were found between the 2 conditions (Figure 3E and Supplemental Figure S3G). We then evaluated whether the contractile apparatus is modified by LOE. To that we measured the sarcomere length in the two differentiated protocols in hiPSC-CMs stained with cardiac troponin T (cTnT). No change in the distance between sarcomere was found (1.86 ± 0.05 µm for LOE vs. 1.84 ± 0.07 µm for HOE, *p* = 0.78) (Figure 3F,G). Similar data were found using the α-sarcomeric actinin staining (Supplemental Figure S2).

### 2.3. LOE Enhances the Expression of the Cardiac Markers in hPSC-CMs in 3D Cardiac Spheroids

**Figure 4:**
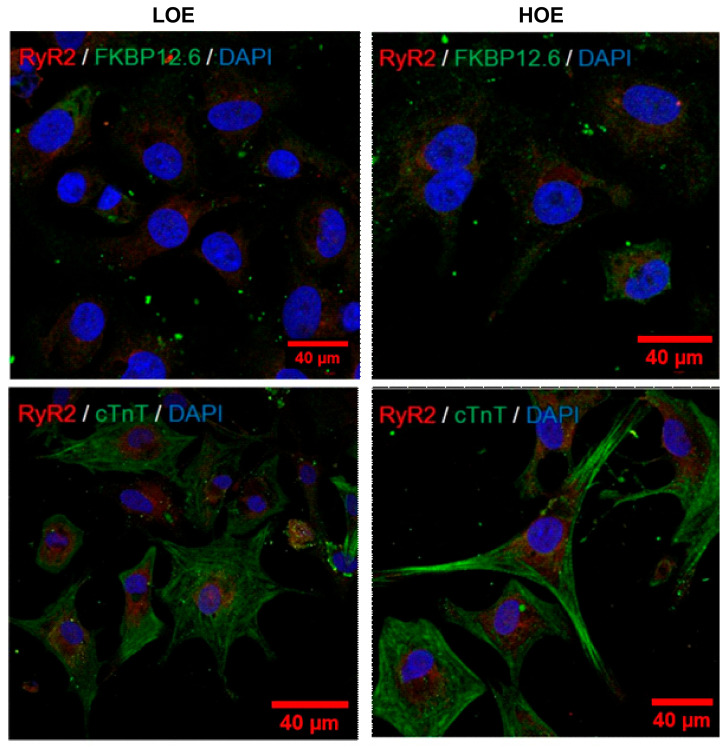
3D cardiac spheroids exhibit cardiac specific markers under both oxygen exposures. Immunostaining for RyR2 (red) and FKBP12.6 (green) (**top**) and cardiac troponin T (cTnT, green) (**bottom**) in 25-day-old hPSC-CMs (using hiPSC UEFhfiPS1.4 and hESC CCTL12 lines) differentiated in LOE or HOE conditions. Nuclei are stained with DAPI (blue). Scale bars: 40 µm.

We evaluated the expression and pattern of a major cardiac protein composing the sarcomeres (cTnT) and the main actor of the intracellular release of Ca^2+^ and its stabilizing partner (ryanodine receptor type 2 or RyR2 and FK506 binding protein or FKBP12.6, respectively) using immunocytochemistry. In both conditions, we found a striated sarcomeric pattern of cTnT and a scattered expression of RyR2 and FKBP12.6 (Figure 4).

**Figure 5:**
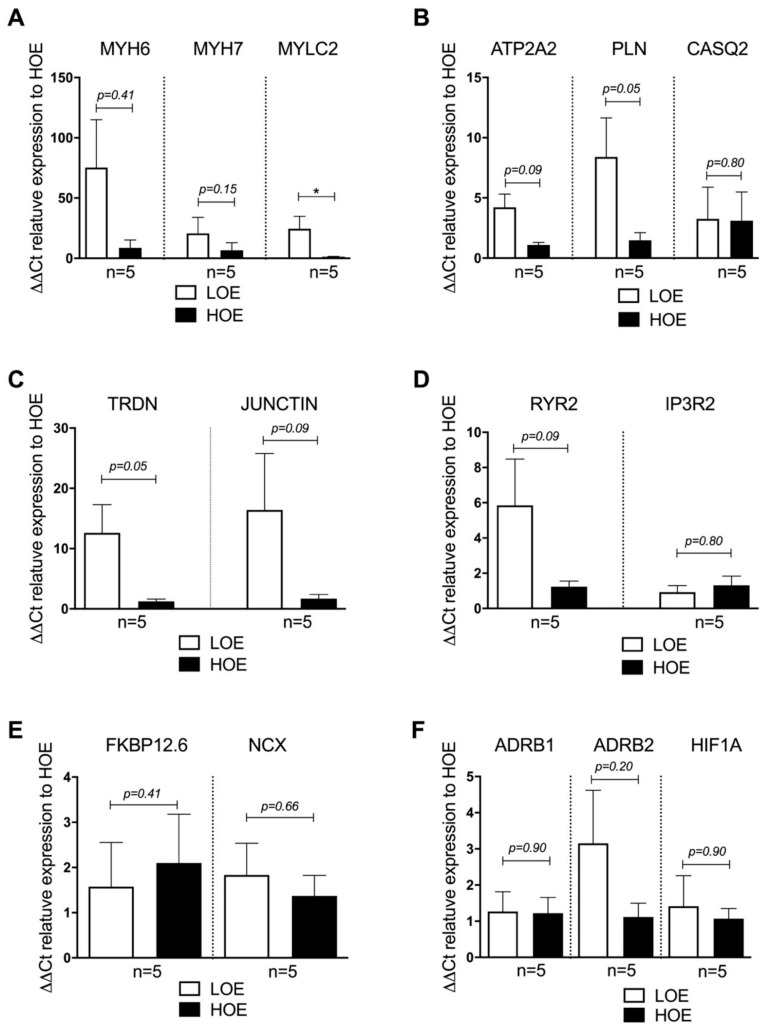
3D cardiac spheroids differentiated under LOE exhibit higher expression in key cardiac markers. Relative gene expression analysis by qRT-PCR in 25-day-old hPSC-CMs (using hiPSC UEFhfiPS1.4 and hESC CCTL12 lines) obtained from 3D cardiac spheroids differentiated in LOE (white bars) and HOE (black bars) conditions. (**A**) MYH6 = alpha-myosin-heavy-chain (*n* = 5); MYH7 = beta-myosin-heavy-chain (*n* = 5); MYL2 = Myosin Light Chain 2 (*n* = 5); (**B**) ATP2A2 = ATPase Sarcoplasmic/Endoplasmic Reticulum Ca^2+^ Transporting 2 (*n* = 5); PLN = phospholamban (*n* = 5); CASQ2 = calsequestrin 2 (*n* = 5); (**C**) RYR2 = ryanodine receptor type 2 (*n* = 5); IP3R2 = inositol 1,4,5-triphosphate type 2 receptor (*n* = 5); (**D**) FKBP12.6 = Calstabin 2 (*n* = 5); NCX = Na^+^ Ca^2+^ exchanger (*n* = 5); (**E**) TRDN = triadin (*n* = 5); JUNCTIN = Junctin (*n* = 5); (**F**) ADRB1 = beta-1 adrenergic receptor (adrenoceptor beta 1) (*n* = 5); ADRB2 = beta-2 adrenergic receptor (adrenoceptor beta 2) (*n* = 5); HIF1A (hypoxia-inducible factor 1-alpha (*n* = 5). Glyceraldehyde-3-phosphate dehydrogenase (GAPDH) was used as housekeeping gene. The number of experiments is 5 independent biological replicates for each bar graph. Data are shown as mean ± SEM. Significance was calculated by Mann-Whitney test. *, *p* < 0.05.

In order to further assess whether the oxygen exposure modulates the cardiac gene expression in 3D spheroids at late stage (i.e., 25-day-old EBs), we performed qRT-PCR experiments. Relative expression analysis indicated high variability in any tested gene. We observed a higher expression of myosin light chain 2 (*MYLC2*) under LOE and compared to HOE (ratio of 24.53 ± 10.33 for LOE vs. 1.30 ± 0.31 for HOE, *p* < 0.05) (Figure 5A). A tendency to a higher expression of typical cardiac markers in hPSC-CMs differentiated in LOE compared to hPSC-CMs differentiated in HOE was also observed, including the *RYR2* gene (ratio of 5.85 ± 2.63 for LOE vs. 1.23 ± 0.31 for HOE, *p* = 0.09), ATPase sarcoplasmic/endoplasmic reticulum Ca^2+^ Transporting 2 (*ATP2A2*) (ratio of 1.53 ± 0.97 for LOE vs. 1.09 ± 0.21 for HOE, *p* = 0.09), triadin (*TRDN*) (ratio of 12.62 ± 4.65 for LOE vs. 1.23 ± 0.37 for HOE, *p* = 0.05) and sodium/calcium exchanger (*NCX*) (Figure 5B–E). The expressions of the β_1_ adrenergic receptor (*ADRB1*) and β_2_ (*ADRB2*) were similar between LOE and HOE, as well as the hypoxia-inducible factor 1 alpha (*HIF1A*) (Figure 5F). To make sure that the reference gene was not affected by the oxygen exposure, we next evaluated the expression of these same genes using another reference gene, namely Ribosomal Protein Lateral Stalk Subunit P0 (*RPLP0*) and obtained the same results (Supplemental Figure S3). It is noteworthy that none of these cardiac genes were expressed in hPSC in LOE and HOE before differentiation (Supplemental Tables S1 and S2).

### 2.4. LOE Leads to a Steadier SR Ca^2+^ Handling in hPSC-CMs Contained in 3D Cardiac Spheroids

**Figure 6:**
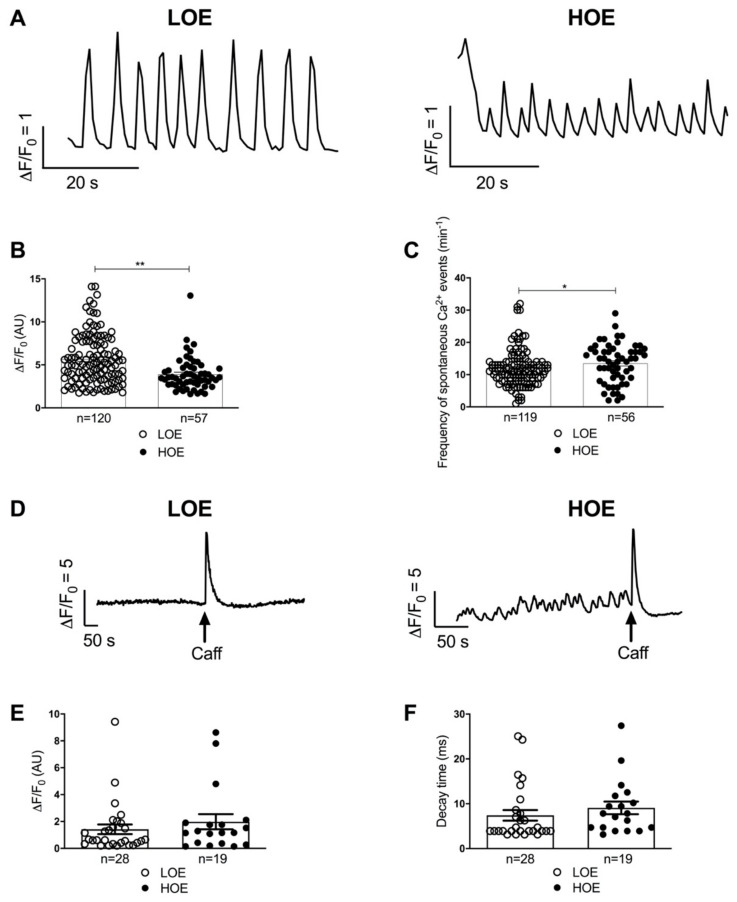
3D cardiac spheroids differentiated under LOE exhibit more mature SR Ca^2+^ handling. Intracellular Ca^2+^ transients were measured in 25-day-old hPSC-CMs (using hiPSC UEFhfiPS1.4 and hESC CCTL12 lines) obtained from 3D cardiac spheroids in LOE (white bars) and HOE (black bars) conditions. (**A**) Display of original corresponding tracing line-scan images of Ca^2+^ in hPSC-CMs differentiated in LOE and HOE conditions. (**B**) Normalized Ca^2+^-transient amplitude in hPSC-CMs differentiated in LOE and HOE conditions. (**C**) Frequency of spontaneous Ca^2+^ events in hPSC-CMs differentiated in LOE and HOE conditions. (**D**) Representative traces of cytosolic Ca^2+^ fluorescence in hPSC-CMs differentiated in LOE and HOE conditions upon 30 mM caffeine (Caff). (**E**) Amplitude of SR Ca^2+^ load in hPSC-CMs differentiated in LOE and HOE conditions. (**F**) Decay phase of the SR Ca^2+^ load in hPSC-CMs differentiated in LOE and HOE conditions. The number of cells evaluated varies from 19 to 120 cells for each dot plot from 3 independent biological replicates. Data are shown as mean ± SEM. Significance was calculated by Mann-Whitney test. *, *p* < 0.05, **, *p* < 0.01.

The intracellular Ca^2+^ cycling is a major component of the cardiac excitation-contraction coupling as the intracellular Ca^2+^ release precedes and initiates the contraction. To assess the impact of oxygen exposure on the intracellular Ca^2+^ cycling of the hPSC-CMs, we evaluated some kinetic properties of the intracellular Ca^2+^ transients in enzymatically dissociated hPSC-CMs from 3D spheroids. Using confocal fluorescent microscopy, we found that 25-day-old hPSC-CMs differentiated under LOE exhibited more regular spontaneous Ca^2+^ transients associated with significant higher maximal amplitude compared to hPSC-CMs differentiated under HOE (5.65 ± 0.26 AU for LOE vs. 3.89 ± 0.25 AU for HOE, *p* < 0.01) (Figure 6A,B). Moreover, hPSC-CMs differentiated in LOE exhibited lower spontaneous Ca^2+^ events (frequency of occurrence of 11.97 ± 0.52 min^−1^ for LOE vs. 13.48 ± 0.81 min^−1^ for HOE, *p* < 0.05) (Figure 6C). We then compared the SR Ca^2+^ content between LOE ad HOE. Application of high concentration of caffeine (30 mM) induced an exhaustive release of SR Ca^2+^ through RyR2 which reflected SR Ca^2+^ content. The release amplitude in response to caffeine (1.42 ± 0.36 AU for LOE vs. 1.98 ± 0.56 AU for HOE, *p* = 0.55) (Figure 6D,E) and the Ca^2+^ reuptake remained unchanged between the 2 groups (Figure 6D,F).

### 2.5. LOE Enhances the Contraction Force in 3D Cardiac Spheroids

**Figure 7:**
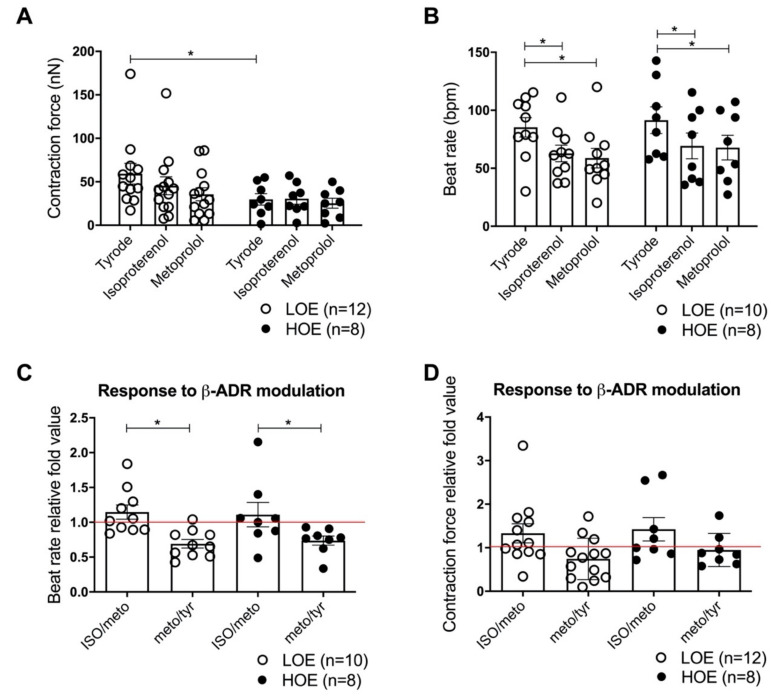
3D cardiac spheroids differentiate under LOE show greater contraction force. Mechanical analysis of contraction force (in nN) and beat rate (in beats/minute, bpm) has been performed using AFM mechanograms in a single position in 3D cardiac spheroids (using hiPSC UEFhfiPS1.4 and hESC CCTL12 lines) differentiated under LOE (white dot plots) and HOE (black dot plots) conditions. (**A**) Contraction force (nN) in 3D cardiac spheroids differentiated under LOE and HOE conditions. (**B**) Beat rate (bpm) in 3D cardiac spheroids differentiated under LOE and HOE conditions. (**C**) Relative analysis of β-adrenergic modulation for beating rate in 3D cardiac spheroids differentiated under LOE and HOE conditions. (**D**) Relative analysis of β-adrenergic modulation for contraction force in 3D cardiac spheroids differentiated under LOE and HOE conditions. 1 mM of isoproterenol was applied during the AFM experiments to stimulate the β-adrenergic receptors. 70 µM of metoprolol was applied during the AFM experiments to inhibit the β1-adrenergic receptors. The number of experiments varies from 8 to 22 3D cardiac spheroids for each dot plot from 3 independent biological replicates. Data are shown as mean ± SEM. Significance was calculated by Mann-Whitney, Kruskal Wallis and 2-way ANOVA tests. *, *p* < 0.05.

We then tested whether the steadier intracellular Ca^2+^ cycling observed in hPSC-CMs differentiated under LOE results in enhanced contractile properties in 3D cardiac spheroids. We evaluated key mechanical properties including the contraction force and beat rate using AFM at 37 °C as we previously published (Pesl et al., 2014, 2016; Acimovic et al., 2018). We observed a stronger contraction force in 3D cardiac spheroids differentiated under LOE (59.32 ± 11.83 nN for LOE vs. 29.77 ± 6.53 nN for HOE, *p* < 0.05) (Figure 7A). No change was observed in the beat rate of cardiac spheroids (85.18 ± 8.33 bpm for LOE vs. 91.47 ± 11.48 bpm for HOE, *p* = 0.9) (Figure 7B). Next, we evaluated the β-adrenergic receptor response to contraction force and beating rate by activating this pathway using the non-specific agonist isoproterenol (ISO) and a selective β_1_-adrenergic receptor antagonist metoprolol (METO). The contraction force response of the 3D cardiac spheroids differentiated in LOE and HOE was similar upon the application of ISO and METO (Figure 7A). Both molecules decreased the beat rate in the 2 types of cardiac spheroids (Figure 7B). The chronotropic and ionotropic effects of ISO were similarly positive in LOE and HOE, while METO induced negative chronotropic and ionotropic effects with no difference between LOE and HOE (Figure 7C,D).

## 3. Discussion

**Figure 8:**
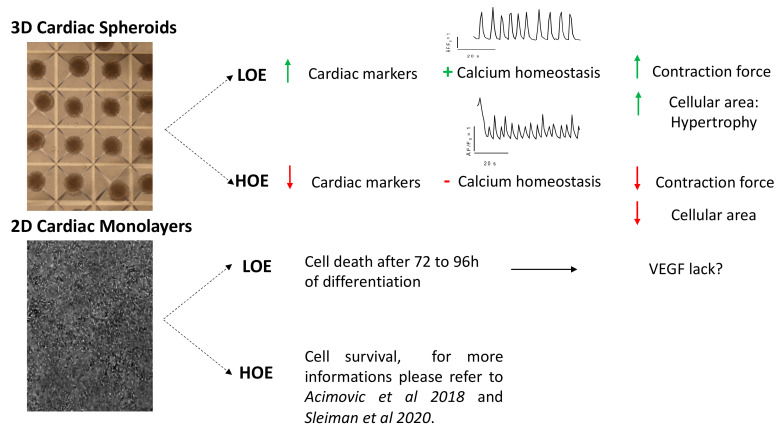
Schematic conclusion representing the main findings. 3D cardiac spheroids differentiated under LOE present a higher expression of cardiac markers, mature Ca^2+^ homeostasis parameters, higher contraction force and increase of cell area compared to 3D cardiac spheroids differentiated under HOE conditions. 2D cardiac monolayers differentiated under LOE die after 72 to 96 h of differentiation.

In this study, we used 3 different hPSC lines to evaluate the impacts of LOE and HOE on the molecular and functional properties of hPSC-CMs differentiated in the 2D monolayers and 3D cardiac spheroids by using 2 of the most commonly used protocols of cardiac differentiation [1,12,13]. Here, we found that LOE (1) abolishes the early cardiac differentiation of 2D monolayers but not 3D spheroids, (2) does not impact the mitochondrial metabolism and O_2_ consumption nor the mitochondrial localization, (3) enhances cardiac hypertrophy and higher molecular cardiac marker expression in hPSC-CMs differentiated in 3D cardiac spheroids, and (4) leads to a steadier SR Ca^2+^ cycling associated with higher contraction force of the 3D cardiac spheroids. These findings, summarized in Figure 8, revealed that O_2_ is an ambivalent parameter and suggested that LOE is a relative optimal factor of differentiation when limited to 3D cardiac spheroids.

Our results suggested that LOE is an incompatible condition for differentiating 2D cardiac monolayers while the 3D cardiac spheroids were successfully differentiated with this condition. In the 2D cardiac monolayers, the hPSC-CMs died after 72 h when compared to those maintained in HOE. The 2D cardiac monolayer differs from 3D EBs by the lack of endoderm (inside layer) and ectoderm (outside layer) which both surround mesoderm. The absence of these 2 layers may explain the massive monolayer mesodermal cell death we observed under LOE. In comparison with the 2D cardiac monolayer, our protocol to obtain 3D cardiac spheroids contained VEGF, which has been shown to promote CM differentiation of mouse PSCs via ERK-mediated Flk-1 and Flt-1 activation [18]. VEGF has a major role in angiogenesis upon heart development [19,20]. Mice lacking VEGF-B have smaller hearts [21]. The absence of VEGF during the differentiation of 2D cardiac monolayers may explain the inability to maintain the hPSC-CMs under LOE. More experiments testing VEGF application on the 2D monolayer-based protocol will be needed to decipher its putative role in LOE. In comparison, the 3D cardiac spheroids were successfully differentiated under both LOE and HOE and the resulting hPSC-CMs were investigated. It is known that EBs can be maintained under LOE by transiently activating the hypoxia-inducible factors (HIFs) [22].

We tested whether the mitochondria were impacted by O_2_ exposure by evaluating the O_2_ consumption through the respiratory chain complexes. For each complex of the mitochondrial respiratory chain, we observed no difference in hPSC-CMs to consume O_2_ between those differentiated in HOE and LOE. In both conditions, our data indicated that hPSC-CMs at an early-stage of differentiation (12-day-old) mostly exhibit mitochondrial glycolysis. These results supported previous findings on the immature mitochondrial metabolism of hPSC-CMs at the early-stage with privileged glycolysis to produce ATP [6,9]. Further studies will be needed to determine if supplementation in fatty acids at an early-stage modulates the oxidative phosphorylation in hPSC-CMs under LOE and HOE as recently suggested [23]. We studied whether the mitochondrial distribution changes in hPSC-CMs under LOE and HOE. Although hPSC-CMs under LOE displayed cellular hypertrophy, we found that hPSC-CMs exhibited akin mitochondrial distribution in both conditions. One explanation might be related to the lack of cell maturity and absence of stimuli promoting sarcomere organization such as tissue engineering [24,25], adapted substrates [26] and hormone supplementation, including thyroid hormones and glucocorticoids [27,28]. Jeziorowska et al. found smaller sarcomere length in hiPSC-CMs differentiated in a 3D compared to 2D protocol [29]. Here, we found that the sarcomere length of hiPSC-CMs is not affected by oxygen exposure, values close to those in adult cardiomyocytes [30]. These results indicate that O_2_ does not play a role in improving sarcomerization. However, the lack of mitochondrial maturity may explain these results. Further experiments are needed to dig out the real impact of O_2_ in the sarcomerization in hiPSC-CMs.

We found no evident effect of LOE to promote cardiac differentiation beside a higher expression of the *MYL2* gene coding for the ventricular myosin regulatory light chain. The other genes investigated indicated a high variability and a tendency to be enhanced under LOE. In particular, *HIF1A* gene expression remained unchanged. This unexpected result might be due to the fact that the EBs were re-incubated in HOE after 12 days of differentiation in LOE. This change might prevent the increased HIF1-α expression under LOE. Still, our results support the role of O_2_ to modulate the mesoderm and early cardiomyogenesis as it was previously suggested through HIF1α-mediated transcriptional regulation of key components of myofibrillogenesis and the cardiac transcription factor network [9]. We observed that LOE favors a steadier SR Ca^2+^ cycling and increases the Ca^2+^ transient amplitude. In line with these observations, we found that LOE also favors higher contraction force in 3D cardiac spheroids. These results match with the cellular hypertrophy under LOE. Overall, our results suggested that LOE is beneficial for the functionality of late-stage 3D cardiac spheroids when compared to those differentiated under HOE. These results support the findings from other groups claiming that hypoxia enhances cardiac differentiation [3,9,31]. However, there was no difference in the β-adrenergic receptor response to contraction force and beating rate between the 3D spheroids differentiated under LOE and HOE. Similar gene expression for the β_1_ and β_2_-adrenergic receptor was also observed. These results suggested that O_2_ does not modulate the functionality of the β-adrenergic receptor pathway of hPSC-CMs at late stage (25-day-old).

Taken all together, our findings indicated that LOE slightly enhances some of the molecular and functional features of the hPSC-CMs. Interestingly, our results indicate that LOE-induced cell improvement is limited to hPSC-CMs differentiated through 3D differentiation. The LOE does not allow differentiation of the 2D cardiac monolayer for which HOE (i.e., 21% O_2_) appears to be crucial.

In conclusion, this work suggests that LOE is an important factor that should be carefully considered in cardiac differentiation. LOE enhances the maturity of hPSC-CMs through 3D EB-based protocol, while it abolishes cardiac differentiation through 2D monolayer-based protocol. This work may contribute to further determining the optimal condition to obtain mature human stem cell-derived cardiomyocytes in vitro.

## 4. Material and Methods

### 4.1. Cell Lines

All subjects gave their informed consent for inclusion before they participated in the study. The study was conducted in accordance with the Declaration of Helsinki, and the protocol (10-02214) was approved by the Ethics Committee of the UCSF Medical Center (CA, USA). Several stem cell lines, including hiPSC *UEFhfiPS1.4* [32], hESC *CCTL12* [13,33,34] and hiPSC *UB47* [12,33], were maintained as colonies on mitotically inactivated mouse embryo fibroblast (MEF) feeder in HES medium with 10 ng/mL human FGF2 or single cells on Matrigel hES-qualified (Corning, Corning, NY, USA, ref: 354277) as previously described [12]. Briefly, the colonies were manually dissected using a needle and passaged every 4–6 days whereas the single cells were enzymatically dissociated using TrypLE enzyme (Gibco, Waltham, MA, USA, ref: 12604-013) and passaged every 4 days.

### 4.2. Cardiac Differentiation

All hPSC lines were differentiated in CMs using both the 2D (monolayer-based) protocol and 3D (EB-based) protocol as we have previously published them [12,13,33]. Briefly, hPSC were enzymatically dissociated by TrypLE enzyme and were transferred to AggreWell 400 plate (Stemcell Technologies, Vancouver, BC, Canada, ref: 34425) to form 3D cardiac spheroids (embryoid bodies, EBs). AggreWell 400 pre-formed dishes, together with the spinning of cells, enable formation of highly and uniformly sized and shaped EBs of about 200 µm in diameter. For the 2D protocol, undifferentiated single hPSCs, enzymatically dissociated by TrypLE enzyme, were plated into 6-well dishes and were differentiated when 90% of confluency was reached (Figure 1). For generating 3D cardiac spheroids from hPSCs using AggreWell 400 plates, 0.5 mL/well of 3D cardiac formation medium (StemPro34, Gibco life technologies, ref: 10639-011) was added. After spinning down at 2000 g for 5 min 2.4 × 10^6^ single hPSCs/well were added. 1.5 mL/well StemPro34 supplemented with 10 µM ROCK inhibitor (Miltenyi Biotec, Bergisch Gladbach, Germany, ref: 130-106-538) was added. After gently pipetting several times to re-establish an even distribution of cells throughout the well, the Aggrewell 400 plates were spun down at 100 g for 3 min. The cells were then incubated either in LOE (5% O_2_ and 5% CO_2_ at 37 °C) using a New Brunswick™ Galaxy^®^ 48 R CO_2_ incubator or HOE (21% O_2_ and 5% CO_2_ at 37 °C) and 95% humidity overnight. For medium change under LOE, medium was pre-incubated in the hypoxic incubator for 30 min. 24 hrs after seeding the cells, formed cardiac spheroids were transferred to low-adhesive 60-mm diameter Petri dishes in induction medium composed of cardiac medium supplemented with 10 ng/mL bone morphogenetic protein 4 (BMP4, R&D, Minneapolis, MN, USA, ref: 314-BP-010), 5 ng/mL FGF2 (Miltenyi, ref: 130-093-837), and 6 ng/mL activin A (R&D Systems, ref: 338-AC-010). At day 5, cardiac progenitor formation was induced by adding StemPro34 with 10 µM of the inhibitor of Wnt response 1 (IWR1) (Sigma, St. Louis, MO, USA, ref: I0161) and 10 ng/mL VEGF (R&D Systems, ref: 293-VE). After day 8, the medium was changed 3 times per week by adding 10 ng/mL VEGF and 5 ng/mL FGF2 each time. For the 2D sandwich-based protocol, undifferentiated single hPSCs were plated into 6-well dishes either in LOE or HOE conditions. At 90% confluency (day 1), 0.04 mg of Matrigel reduced growth factor (MgFr) (Corning, ref: 354230) was added in Stemflex medium (Gibco, 75 A33493-01). On day 0, mesoderm was induced by adding 6 μM CHIR99021 (Calbiochem, ref: 3615715) and 0.04 mg of MgFr in RPMI 1640-B27-minus insulin medium (Gibco, ref: 21875-034). On day 2, the medium was changed with RPMI 1640-B27-minus insulin. On day 3, cardiac progenitor formation was induced by adding 2 μM Wnt inhibitor C59 (Wnt C59) (Tocris, Bristol, UK, ref: 5148/10) in RPMI 1640-B27-minus insulin; the medium was kept for 2 days. On day 5, the medium was changed to RPMI 1640-B27-minus insulin and was kept until day 9 and then renewed every 2 days. On day 9, the medium was changed to RPMI 1640-B27 and was kept until day 30; the medium was renewed every 2 days. It should be noted that the LOE was only used to differentiate the hPSC into CMs during the first 12 days of differentiation for cardiac spheroids, and for the first 9 days for the 2D monolayer thereafter, the CMs were kept in HOE.

The duration of 12 and 9 days, respectively, were chosen based on our previous published work [12,13,33]. These days correspond to the time required for obtaining beating hPSC-CMs. For cell viability experiments, the 2D-monolayer cells were washed 3 times with Ca^2+^- and Mg^2+^-free PBS (Sigma, ref: D8537). The cells were dissociated by incubating them for 10 min at 37 °C with pre-warmed TrypLE (Gibco) with periodic shaking. RPMI 1640-B27 was added to stop the activity of TrypLE. The viable cells were then counted using Malassez cell counting chamber.

### 4.3. qRT-PCR

25-day-old beating EBs were used for RNA extraction. Total RNA was isolated using a NucleoSpin RNA kit (Macherey-Nagel, Düren, Germany, ref: 740955.50) followed by reverse transcription of 1 μg total RNA into cDNA that was achieved using the Transcriptor Universal cDNA Master kit (Roche, Basel, Switzerland, ref: 05893151001), according to manufacturers’ protocols. For the detecting of cardiac markers, such as cardiac ion channel expression and regulation, and cardiomyocyte structure, the cDNA was amplified by LightCycler 480 SYBR Green I Master (Roche, ref: 04707516001) for Quantitative Polymerase Chain Reactions (qPCR) that were executed in quadruplicates. Glyceraldehyde-3-Phosphate Dehydrogenase (*GAPDH*) and Ribosomal Protein Lateral Stalk Subunit P0 (*RPLPO*) were used as 2 reference genes. Primers for relative human cardiac marker expression are listed in a previously published study [12], except the Adrenoceptor Beta 1 (*ADRB1*) primers (Forward: CCAGAAGCAGGTGAAGAAGAT, Reverse: CAGCCAGTTGAAGAAGACGAA) and Adrenoceptor Beta 2 (*ADRB2*) primers (Forward: CTCCCAGGCACGGAAGA, Reverse: CCTCCCTTGTGAATCAATGTT), Natriuretic Peptide B (*BNP*) primers (Forward: TTGGAAACGTCCGGGTTAC, Reverse: GGACTTCCAGACACCTGTGG), Actin Alpha Skeletal Muscle 1 (*ACTA1*) primers (Forward: CACAATGTGCGACGAAGACG, Reverse: ATGATGCCGTGCTCGATAGG), and Hypoxia-inducible factor 1-alpha (*HIF1A*) primers (Forward: CCATTAGAAAGCAGTTCCGC, Reverse: TGGTAGTGGTGGCATTAGC). The data were processed using the LightCycler 480 software. Genes with (Cycles threshold) Ct > 35 were considered undetectable. The ΔCt was obtained by normalizing the mean expression values of each gene to the reference genes. The ΔΔCt was calculated by normalizing to the high oxygen exposure (HOE).

### 4.4. Embryoid Body Dissociation

Prior to the staining of cardiac markers, beating (contracting) EBs were enzymatically dissociated in order to isolate the CMs. EBs were first collected and washed two times in Ca^2+^-free solution (120 mM NaCl, 5.4 mM KCl, 5 mM MgSO_4_, 5 mM sodium pyruvate, 20 mM glucose, 20 mM taurine, and 10 mM HEPES; pH 6.9). EBs were then spun down at 15 g for 3 min, and the Ca^2+^-free solution was exchanged for the second time. The EBs were left for 20 min at room temperature before centrifugation. After spinning down and removing the supernatant, EBs were incubated for 3–5 min at 37 °C in digestion solution (Ca^2+^-free solution supplemented with 0.8 mg/mL type II collagenase from *Clostridium histolyticum* (ThermoFisher, ref: 17101015) and 0.04 mg/mL type XIV protease from *Streptomyces griseus* (Sigma, ref: p5147) pre-warmed to 37 °C, with periodic shaking. After spinning down at 170 g for 3 min, the pellet was resuspended in 1 mL of Kraft-Bruhe (KB) solution (85 mM KCl, 30 mM K_2_HPO_4_, 1 mM EGTA, 2 mM ATP-Na_2_, 5 mM sodium pyruvate, 5 mM creatine, 20 mM taurine, and 20 mM glucose; pH 7.3), pre-warmed to 37 °C, and incubated for 20 min at 37 °C, with periodic shaking. After spinning down at 170 g for 3 min, the pellet was gently resuspended in 100 µL of StemPro34 medium. Finally, the CMs were plated on the gelatin-coated 12 mm diameter coverslips in the StemPro34 medium.

### 4.5. Immunocytochemistry

For detection of cardiac markers, namely cardiac ryanodine receptor (RyR2) and FK506 binding protein (FKBP12.6), dissociated beating EBs were fixed with 4% PFA for 1 h on ice, washed with 1× PBS, permeabilized with 0.25% Triton in 1× PBS for 10 min at room temperature, and blocked with 1% bovine serum albumin (BSA) (Sigma ref: A2153) in 1× PBS for 30 min at room temperature. Incubation with primary antibodies RyR2 (Millipore, ref: AB9080) and FKBP12.6 (Santa Cruz, ref: sc-131520) and troponin T-C (C-19) (cTnT, ThermoScientific, ref: MA5-12960) diluted in 1% BSA in 1× PBS was at 4 °C, overnight, and the next day, with secondary antibody Alexa secondary antibodies (ThermoFisher, ref: A1101) for 1 h at room temperature. After that samples were mounted in Mowiol (Sigma ref: 81381) with 4,6-diamidino-2-phenylindole (DAPI; Sigma, ref: 81386). Images were collected on an inverted confocal fluorescent microscope equipped with Zeiss LSM780 confocal and 63x lens (oil immersion, N.A. = 1.4) with Zen software.

### 4.6. Measurement of Cytosolic Ca^2+^ Variations

Measurement of cytosolic Ca^2+^ variations were performed as previously published [12]. After dissociation of 25-day-old contracting EBs, hPSC-CMs were loaded with 3 µM Fluo-4 AM Ca^2+^ indicator (Thermo Fisher ref: F-14201) in Tyrode’s solution for 20 min at RT. Cells were placed in an experimental chamber on the stage of an inverted microscope. Ca^2+^ line scans were recorded with an inverted confocal microscope (Zeiss LSM Exciter) equipped with a 40x lens (water immersion, numerical aperture, N.A. = 1.4). Confocal images were obtained in plane (frame) scan mode at a rate of 1 image/0.495 s at room temperature. The caffeine experiments were used to measure the SR Ca^2+^ load in Tyrode solution. 30 mM caffeine (Sigma, ref: C0750) was added during the acquisition. The acquisition was performed in plane (frame) scan mode in x-y mode at a rate of 1 image/0.782 s. To enable comparisons between cells, changes in the Fluo-4 fluorescence signal (ΔF) were normalized by basal fluorescence (F_0_) as previously performed [33]. All experiments were performed at room temperature. All data were extracted using AIM 4.2 (Zeiss). Maximal amplitudes and event frequencies were extracted and analyzed from raw data by an in-house developed algorithm implemented in Python (version 3.0) (https://asalykin.github.io/PeakInspector/) and GraphPad Prism (version 7.0) as previously done [12,33].

### 4.7. Atomic Force Microscopy (AFM)

AFM experiments on EBs were performed as previously published [12,13,34,35]. Twenty-five-day-old homogenous EBs in size and shape were plated on the gelatin-coated 40 mm diameter Petri dishes (TPP) in MEF medium (KO-DMEM with 10% heat-inactivated FBS, 1% L-glutamine, 1% non-essential amino acids, 1% penicillin-streptomycin, 0.1 mM β-mercaptoethanol) for 48 h prior the AFM experiments. MEF medium was exchanged for Tyrode’s solution (140 mM NaCl, 4 mM KCl, 1 mM MgCl_2_, 5 mM HEPES, 10 mM glucose, and 1.8 mM CaCl_2_) 10 min prior to each measurement. JPK Nanowizard 3 (JPK) BioAFM microscope placed on Olympus IX81 (Olympus corporation) inverted optical microscope was used for measurements. Uncoated silicon nitride AFM cantilevers HYDRA2R-50N from Applied NanoStructures (Mountain View) were used for all experiments. All the probes were calibrated prior to the biomechanical experiments. The sensitivity of AFM setup was calibrated by force-distance curve measurement (setpoint value 1.5 V, lifting height 600 nm), giving typical values between 25.5 and 32.5 nm/V. The stiffness of the cantilever was subsequently calibrated by thermal noise measurement, typical values found here were lying between 0.061 and 0.090 N/m. AFM cantilever was placed over the EB surface by motorized stage allowing X-Y movement of the dish, when the situation was monitored with an optical microscope. The cantilever was afterwards introduced into the constant contact with EB surface, with following feedback loop parameters: setpoint 6.0 nN, iGain 0.01 and pGain 50. The feedback loop function principle is to keep the cantilever-EB interaction force constant at 6 nN by force compensation, which is shown in oscilloscope window as mechanocardiogram (MCG). Recorded data sets were evaluated using an in-house developed algorithm, as previously published [12,13,34,35].

### 4.8. Determination of the Activity of the Respiratory Chain Complexes

The mitochondrial respiration in hPSC-CMs was measured and analyzed by means of the high-resolution respirometry using an O_2_K-Oxygraph (Oroboros Instruments). The cells were put into the chambers in Mir05 solution (0.5 mM EGTA, 3 mM MgCl2.6H2O, 60 mM K-Lactobionate, 20 mM Taurine, 10 mM KH2PO4, 20 mM HEPES, 110 mM Sucrose, 1 g/L BSA (pH = 7.1)) and continuously stirred at 37 °C. For permeabilization of the cell membrane, 5 mg/mL digitonin (Sigma, ref: D141) was added. Mitochondrial respiration was quantified by adding complex I (5 mM malate (Sigma, ref: M8304) and 5mM pyruvate (Sigma, ref: P2256)), 1.5 mM ADP (Sigma, ref: A2754), 8μM cytochrome C (Sigma, ref: C3131), and complex II (10 mM succinate (Sigma, ref: S9512)) substrates in order to obtain the maximal mitochondrial respiration driven by complex I and II. Ten μM rotenone (Sigma, ref: R8875) was added to inhibit the complex I in order to obtain the respiration driven by complex II. Then, 8 μg/mL oligomycin (Sigma, ref: O4876) was added to inhibit the ATP synthase in order to obtain the LEAK-respiration state driven by complex II as an indicator of mitochondrial coupling. This step was followed by the addition of 1 mM of the uncoupler FCCP (Sigma, ref: C2920) in order to test the quality of the permeabilization of the plasma membrane by digitonin. At the end of the experiment, the content of the chambers was transferred (2 × 800 μL per chamber) to Eppendorf’s tubes and centrifuged for 5 min at maximum speed. The supernatant was removed, and the pellets were stored at −20 °C or resuspended directly to perform a protein assay using the BCA kit. The data were analyzed using Datlab software (Oroboros Instruments). The flux values (nmol/min) were normalized to the corresponding pellet protein assay concentration to obtain oxygen consumption in nmol/min/mg protein.

### 4.9. Mitotracker

Mitochondria staining was performed using MitoTracker Red CMXRos (Molecular Probes, Eugene, OR, USA, M-7512). After EBs dissociation, derived cardiomyocytes were incubated with 250 nM MitoTracker in MEF medium for 30 min at 37 °C. Then, cells were washed five times with MEF medium. Each washing step was performed for 5 min at 37 °C. Then, cells were washed once with 1× PBS and fixed with 4% PFA for one hour on ice. After washing of cells with 1× PBS, staining for a cardiac marker cTnT was performed. Firstly, the cells were incubated with 1% BSA, 0.1% Triton in 1× PBS for 15 min at room temperature in the dark. After washing with 1× PBS for 5 min, the cells were incubated with 1% BSA, 0.03% Tween, in 1× PBS for one hour at room temperature in the dark. Incubation with the cTnT diluted (1:250) in 0.05% Tween in 1× PBS took place overnight at 4 °C. Next day, the cells were washed five times, 5 min each wash, with 0.05% Tween in 1× PBS at room temperature. Subsequently, the cells were incubated with the secondary antibody Alexa Fluor 488 donkey anti-goat (Invitrogen, Carlsbad, CA, USA, A-11055; dilution 1:500) together with DAPI (dilution 1:1000) in 1× PBS for one hour at room temperature in the dark. After washing of cells with 1× PBS five times, samples were mounted in Mowiol.

### 4.10. Measurement of Cell Areas and Sarcomere Length

The cell area measurement was performed in single-blind by manually outlining the cells with FIJI-ImageJ (https://imagej.net/Fiji/Downloads). Measurement of the sarcomere length was performed in single-blind by tracing a line of 17 µm across the sarcomeres using an in-house developed macro on FIJI-ImageJ (https://github.com/MontpellierRessourcesImagerie/imagej_macros_and_scripts/wiki/Distance_Between_Minima_Tool).

### 4.11. Statistical Analysis

Normality was tested using the Shapiro–Wilk test. An unpaired *t* test was used to compare 2 independent groups with parametric distribution. A Mann–Whitney test was performed for comparing 2 independent groups with non-parametric distribution. Concerning the AFM experiments, statistical differences were evaluated by two-way ANOVA with Sidak’s post hoc multiple comparison test. For the relative fold value, significance was calculated by Wilcoxon test. All data were expressed as mean ± SEM. A value of *p* < 0.05 was considered significant. *, *p* < 0.05, **, *p* < 0.01, *** otherwise specified. Data analysis and statistics were done with Prism (GraphPad).

## Supplementary Materials

The following are available online at https://www.mdpi.com/1422-0067/22/2/662/s1, **Figure S1**: Representative images of 2D cardiac monolayer differentiated in LOE and HOE using different cell lines, **Figure S2**: Measurement of the sarcomere length of hPSC-CMs stained with α-actinin (in red) and DAPI (in blue) (using hiPSC UEFhfiPS1.4 and hESC CCTL12 lines) in LOE and HOE conditions. **Figure S3**: 3D cardiac spheroids differentiated under LOE exhibit higher expression in key cardiac markers. ΔΔCt relative gene expression analysis by qRT-PCR in 25-days-old hPSC-CMs (using hiPSC UEFhfiPS1.4 and hESC CCTL12 lines) obtained from 3D cardiac spheroids differentiated in LOE (white bars) and HOE (black bars) conditions, **Table S1**: Summary table of Ct relative gene expression analysis by qRT-PCR in hPSC (using hiPSC UEFhfiPS1.4 and hESC CCTL12 lines), **Table S2**: Summary table of Ct relative gene expression analysis by qRT-PCR in hPSC (using hiPSC UEFhfiPS1.4 and hESC CCTL12 lines).
