# Supplementary material for: Oxygen Is an Ambivalent Factor for the Differentiation of Human Pluripotent Stem Cells in Cardiac 2D Monolayer and 3D Cardiac Spheroids
Source: Int J Mol Sci. 2021 Jan 11;22(2):662. doi: 10.3390/ijms22020662 (PMC7827232; doi:10.3390/ijms22020662)
Supplement: Supplementary file 1 [file ijms-22-00662-s001.pdf]

Supplemental online data:

Figure S1. Representative images of 2D cardiac monolayer differentiated in LOE and HOE using different cell lines. (A) Representative images using UEFhfiPS1.4 hiPSC of 2D cardiac monolayer differentiation in LOE (in the top panel) and HOE (in the bottom panel) at day 0 (D0) and day 5 (D5). **(B)** Representative images using hESC CCTL12 of 2D cardiac monolayer differentiation in LOE (in the top panel) and HOE (in the bottom panel) at day 0 (D0) and day 5 (D5). The acquisition was performed using EVOS XL Core Imaging System microscopy (magnification *x4*).

Figure S2. Measurement of the sarcomere length of hPSC-CMs stained with α-actinin (in red) and DAPI (in blue) (using hiPSC UEFhfiPS1.4 and hESC CCTL12 lines) in LOE and HOE conditions. (A) Representative images of hPSC-CMs stained with α-actinin (in red) and DAPI (in blue) in LOE (upper image) and HOE (lower image). The sarcomere length was measured by tracing a line of 17μM across the sarcomeres using an in-house developed macro on Fiji-Image J. Longitudinal plots were obtained through the translation of the fluorescence intensity across the line. **(B)** Distance between sarcomere in hPSC-CMs (using hiPSC UEFhfiPS1.4 and hESC CCTL12 lines) differentiated in LOE and HOE conditions. The number of cells evaluated is 35 for each condition from 3 independent biological replicates. Data are presented as mean ± SEM. Significance was calculated by Mann-Whitney test.

Figure S3. 3D cardiac spheroids differentiated under LOE exhibit higher expression in key cardiac markers. ΔΔCt relative gene expression analysis by qRT-PCR in 25-days-old hPSC-CMs (using hiPSC UEFhfiPS1.4 and hESC CCTL12 lines) obtained from 3D cardiac spheroids differentiated in LOE (white bars) and HOE (black bars) conditions. (A) *MYH6* = alpha-myosin-heavy-chain (n=5); *MYH7* = beta-myosin-heavy-chain (n=5); *MYL2* = Myosin Light Chain 2 (n=5); **(B)** *ATP2A2* = ATPase Sarcoplasmic/Endoplasmic Reticulum Ca^{2+} Transporting 2 (n=5); *PLN* = phospholamban (n=5); *CASQ2* = calsequestrin 2 (n=5); **(C)** *RYR2* = ryanodine receptor type 2 (n=5); *IP3R2* = inositol 1,4,5-triphosphate type 2 receptor (n=5); **(D)** *FKBP12.6*= Calstabin 2 (n=5); *NCX* = Na^+ Ca^{2+} exchanger (n=5); **(E)** *TRDN*= triadin (n=5); *JUNCTIN* = Junctin (n=5); **(F)** *ADRB1* = beta-1 adrenergic receptor (adrenoceptor beta 1) (n=5); *ADRB2* = beta-2 adrenergic receptor (adrenoceptor beta 2) (n=5) ; *HIF1A* = hypoxia-inducible factor 1-alpha (n=5); **(G)** BNP = natriuretic peptide B (n=5) and ACTA1= actin alpha skeletal muscle (n=5) 1. Ribosomal Protein Lateral Stalk Subunit P0 (*RPLP0*) was used as housekeeping gene. The number of experiments is 5 independent biological replicates for each bar graph. Data are shown as mean ± SEM. Significance was calculated by Mann-Whitney test. **, $p < 0.05$.

Tables:

Table S1. Summary table of Ct relative gene expression analysis by qRT-PCR in hPSC (using hiPSC UEFhfiPS1.4 and hESC CCTL12 lines): *MYH7* = beta-myosin-heavy-chain (n=5) ; *RYR2* = ryanodine receptor type 2 (n=5) ; *MYH6* = alpha-myosin-heavy-chain (n=5) and *MYL2* = Myosin Light Chain 2 (n=5). Ribosomal Protein Lateral Stalk Subunit P0 (*RPLP0*) was used as housekeeping gene.

Table S2. Summary table of Ct relative gene expression analysis by qRT-PCR in hPSC (using hiPSC UEFhfiPS1.4 and hESC CCTL12 lines): *MYH7* = beta-myosin-heavy-chain (n=5) ; *RYR2* = ryanodine receptor type 2 (n=5) ; *MYH6* = alpha-myosin-heavy-chain (n=5) and *MYL2* = Myosin Light Chain 2 (n=5). Glyceraldehyde-3-phosphate dehydrogenase (*GAPDH*) was used as housekeeping gene.

Figure S1.

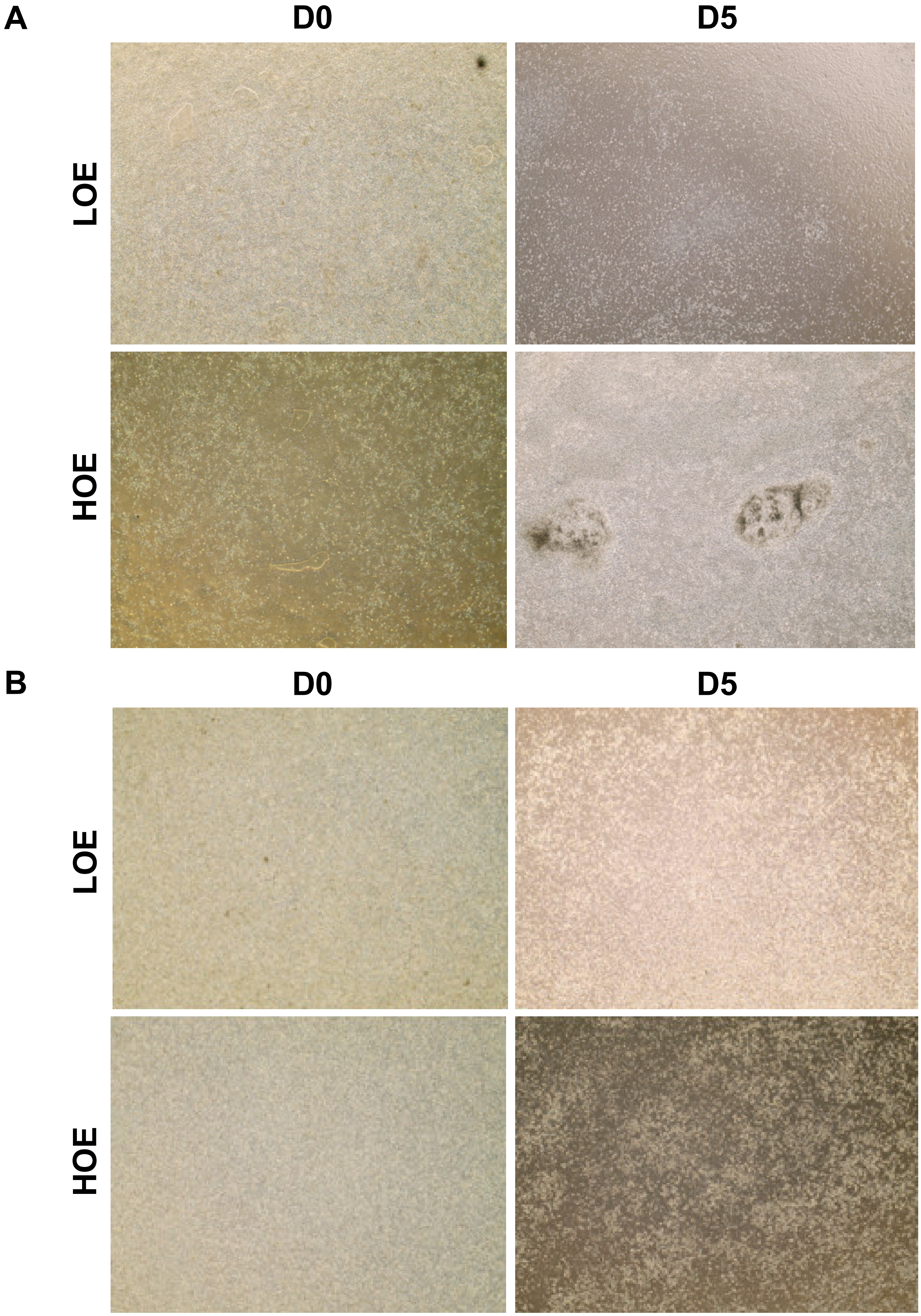

Figure S2.

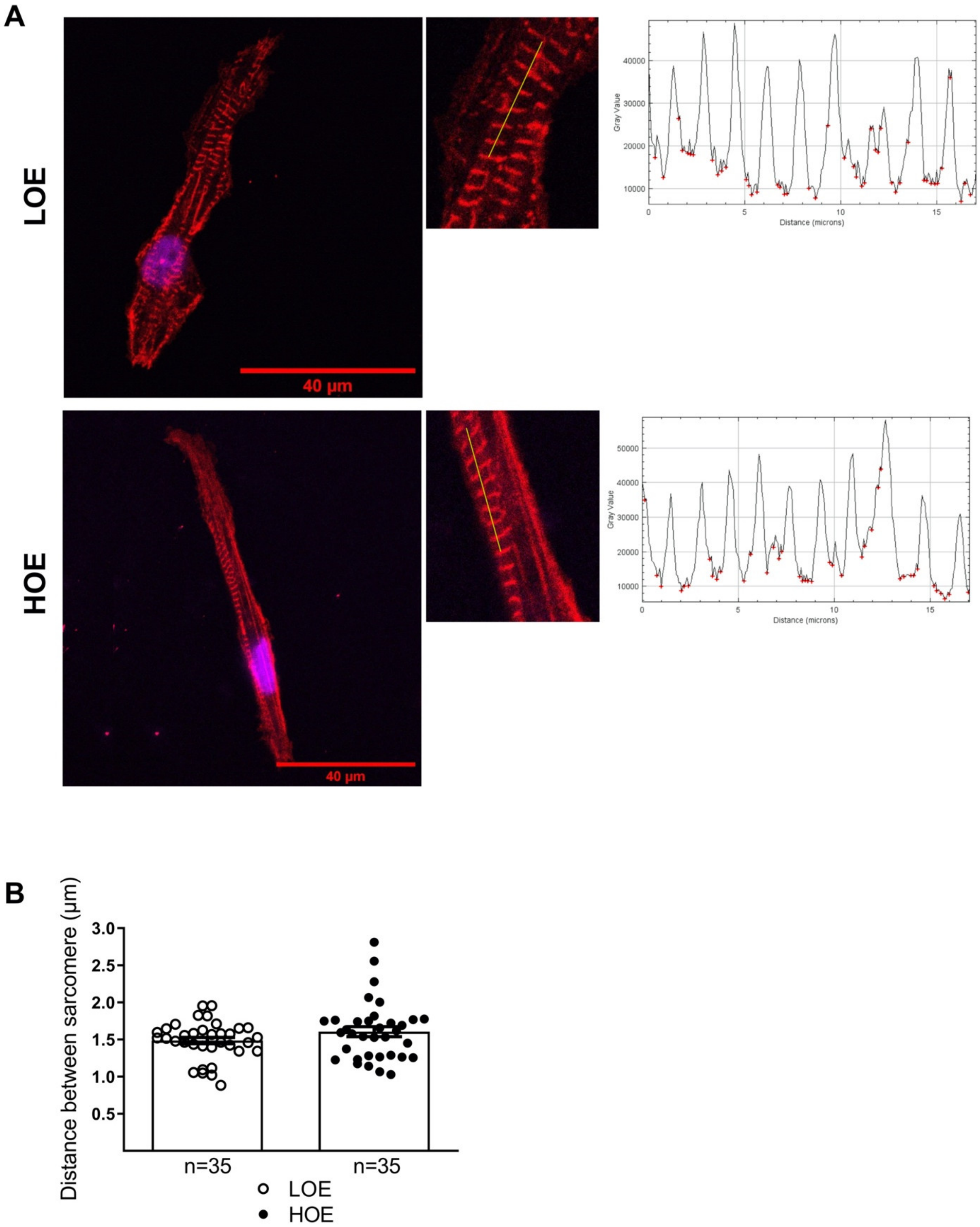
A
LOE
40 µm
Gray Value
Distance (microns)
HOE
40 µm
Gray Value
Distance (microns)
B
Distance between sarcomere (µm)
3.0
2.5
2.0
1.5
1.0
0.5
n=35
n=35
LOE
HOE

Figure S3.

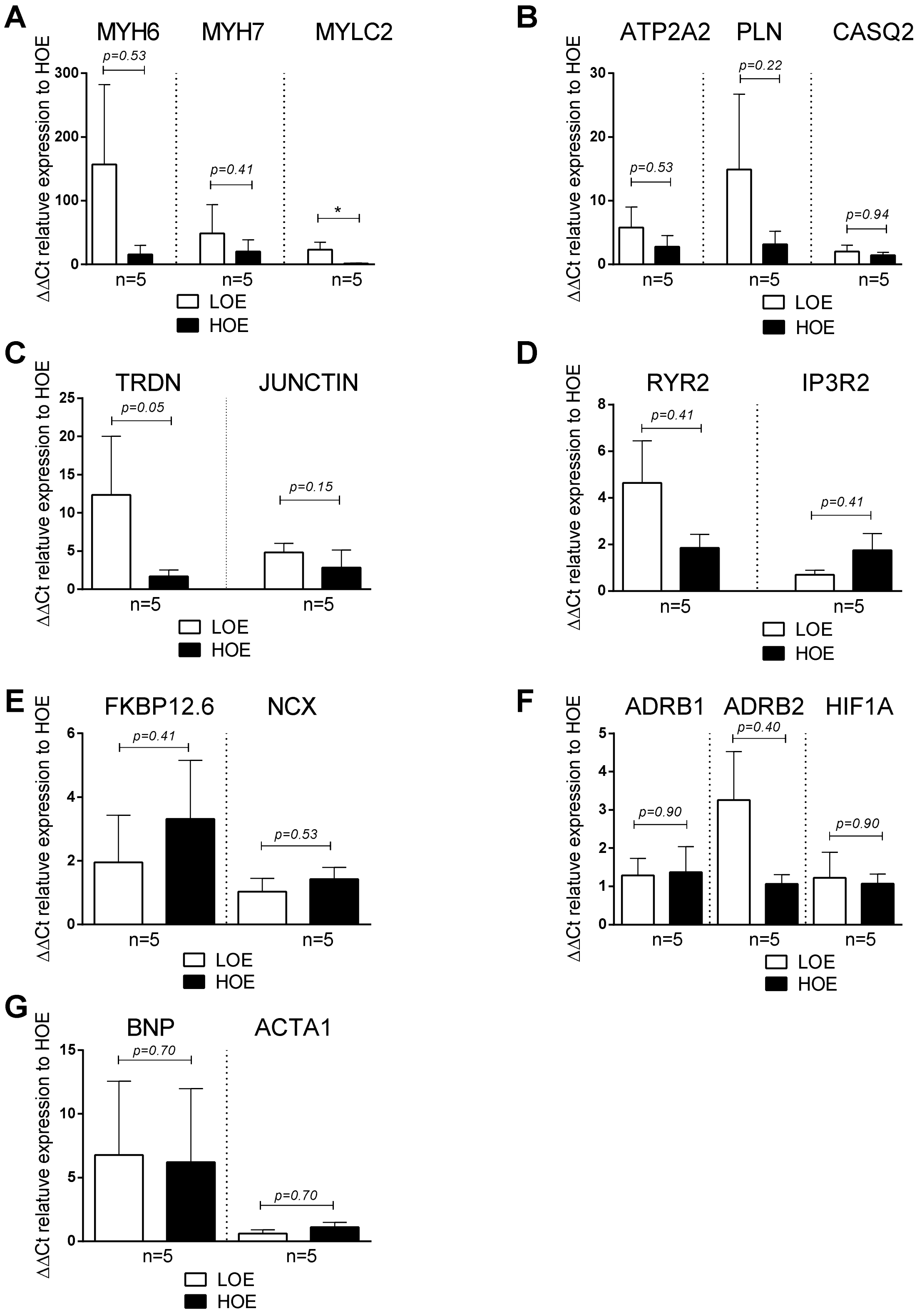
A
MYH6
MYH7
MYLC2
p=0.53
p=0.41
*
300
200
100
0
ΔΔCt relatuve expression to HOE
n=5
n=5
n=5
LOE
HOE
B
ATP2A2
PLN
CASQ2
p=0.53
p=0.22
p=0.94
30
20
10
0
ΔΔCt relatuve expression to HOE
n=5
n=5
n=5
LOE
HOE
C
TRDN
JUNCTIN
p=0.05
p=0.15
25
20
15
10
5
0
ΔΔCt relatuve expression to HOE
n=5
n=5
LOE
HOE
D
RYR2
IP3R2
p=0.41
p=0.41
8
6
4
2
0
ΔΔCt relatuve expression to HOE
n=5
n=5
LOE
HOE
E
FKBP12.6
NCX
p=0.41
p=0.53
6
4
2
0
ΔΔCt relatuve expression to HOE
n=5
n=5
LOE
HOE
F
ADRB1
ADRB2
HIF1A
p=0.90
p=0.40
p=0.90
5
4
3
2
1
0
ΔΔCt relatuve expression to HOE
n=5
n=5
n=5
LOE
HOE
G
BNP
ACTA1
p=0.70
p=0.70
15
10
5
0
ΔΔCt relatuve expression to HOE
n=5
n=5
LOE
HOE

Table S1.

hIPSC / RPLPO					
		Ct gene	delta delta Ct	$2^{-\text{delta delta Ct}}$	Mean HOE Delta Ct
MYH7 LOE	n°1	30,69	15,77	0,07969	14,1205556
	n°2	31,52	14,22	0,23416	
	n°3	31,345	15,45	0,09983	
RYR2 LOE	n°1	21,455	6,54	- 0,80	9,33666667
	n°2	23,535	6,23	-1,11	
	n°3	23,673	7,77	0,44000	
MYH6 LOE	n°1	28,485	0,89	0,53837	14,6716667
	n°2	31,97	1,99	0,25116	
	n°3	33,36	4,79	0,03619	
MLC2 LOE	n°1	29,27	-3,72	13,19269	20,0716667
	n°2	30,335	-5,04	32,93767	
	n°3	30,71	-3,26	9,5909	
MYH7 HOE	n°1	29,845	-0,97	1,95282	
	n°2	30,735	0,98	0,50718	
	n°3	29,667	-0,01	1,00967	
RYR2 HOE	n°1	25,375	-0,65	1,57098	
	n°2	24,825	-0,15	1,10701	
	n°3	25,695	0,8	0,57501	
MYH6 HOE	n°1	31,995	0,63	0,64469	
	n°2	30,015	-0,29	1,22405	
	n°3	29,89	-0,34	1,26722	
MLC2 HOE	n°1	35,87	-0,89	1,85532	
	n°2	34,64	-1,07	2,09459	
	n°3	37,59	1,96	0,25733	

Table S2.

hIPSC / GAPDH					
		Ct gene	delta delta Ct	$2^{-delta\ delta\ Ct}$	Mean HOE Delta Ct
MYH7 LOE	n°1	30,69	15,77	0,07969	12,1205556
	n°2	31,52	14,22	0,23416	
	n°3	31,345	15,45	0,09983	
RYR2 LOE	n°1	21,455	6,54	- 0,80	7,33666667
	n°2	23,535	6,23	-1,11	
	n°3	23,673	7,77	0,44000	
MYH6 LOE	n°1	28,485	0,89	0,53837	12,671667
	n°2	31,97	1,99	0,25116	
	n°3	33,36	4,79	0,03619	
MLC2 LOE	n°1	29,27	-3,72	13,19269	18,07166667
	n°2	30,335	-5,04	32,93767	
	n°3	30,71	-3,26	9,5909	
MYH7 HOE	n°1	29,845	0,28	0,82296	
	n°2	30,735	1,77	0,29366	
	n°3	29,667	-2,05	4,13787	
RYR2 HOE	n°1	25,375	0,6	0,66204	
	n°2	24,825	0,64	0,64097	
	n°3	25,695	-1,24	2,35653	
MYH6 HOE	n°1	31,995	1,88	0,27168	
	n°2	30,015	0,5	0,70874	
	n°3	29,89	-2,38	5,19335	
MLC2 HOE	n°1	35,87	0,35	0,78187	
	n°2	34,64	-0,28	1,21279	
	n°3	37,59	-0,08	1,05458	
